# Comparison of Butyric acid concentrations in ordinary and probiotic yogurt samples in Iran

**Published:** 2012-06

**Authors:** N Vaseji, N Mojgani, C Amirinia, M Iranmanesh

**Affiliations:** 1 Biotechnology Department, National Research Institute of Animal Science, Karaj; 2 Biotechnology Department, Razi Vaccine and Serum Research Institute, Karaj; 3 Department of Food Science and Technology, Science and Research Branch, Islamic Azad University, Tehran, Iran

**Keywords:** Probiotic, butyric acid, yogurt, gas chromatography, starter cultures

## Abstract

**Background and objectives:**

Butyric acid has many applications in chemical, food and pharmaceutical industries. Applications of butyric acid are as an additive to food, flavorings, varnishes, perfumes, pharmaceuticals and disinfectants. Butyric acid concentrations have positive impact on the quality control of milk, yogurt and other probiotic dairy products. The present investigation was undertaken to determine and compare the concentrations of butyric acid (C4) in the ordinary and probiotic yogurt samples by GC method.

**Materials and Methods:**

Probiotic yogurt samples were prepared under laboratory scale conditions using two different commercial starters ABY1 and 211, while ordinary yogurt samples lacked the probiotic starter cultures. All samples were analyzed in duplicate, for C4 concentrations by gas chromatography after day 1, 2, 10 and 20 of production, during storage at 4°C. The results were analyzed using ANOVA and Duncan test.

**Results:**

The level of the mentioned fatty acid in ABY1 yogurt sample was significantly higher (0.2%) than in 211 samples (0.17%). These values were significantly lower in ordinary yogurt samples and only 0.07% was recorded in these samples on first day of storage which decreased gradually during storage. The level of reduction in the yogurt samples tested during different time intervals was not similar in all the examined samples, and some showed enhanced reduction than other samples.

**Conclusions:**

Compared to ordinary yogurt samples, probiotic yogurt samples used in study showed higher levels of butyric acid with increased shelf life.

## INTRODUCTION

In addition to vitamins, calcium, other minerals, and proteins obtained from milk products, modern research has suggested healthful properties of fermentation-derived peptides and butyric acid found in some dairy products (1, 2, 3). Moreover, organic acids are relevant in dairy products for nutritional reasons and because they contribute to the flavor and aroma. They are the major products of carbohydrate catabolism of lactic acid bacteria and nonstarter bacteria associated with milk (4).

Butyric acid has recently been the subject of intensive research due to its purported anti-colon cancer effects. It has also been shown to inhibit the growth of a range of cancer cells. Productions of butyric acid by some probiotic bacteria have been reported. Butyric acid producing probiotic bacteria have been shown to affect the turnover of enterocytes and neutralize the activity of dietary carcinogens, such as nitrosamines, that are generated by the metabolic activity of commensal bacteria in subjects consuming a high-protein diet (5). Therefore, inclusion of probiotic bacteria in fermented dairy products enhances their value as better therapeutic functional foods (6). Butyric acid is also produced synthetically, through fermentation of various carbohydrates, to be used as a flavoring agent in various food products (7).

According to the reports of Heiter and his colleagues (8), the content of butyric acid in milk fat varies ranging between 3 and 4.6%, and for the quality control of milk, yogurt and foodstuffs with additives involving milk and butter the analysis of the content is utilized.

The present research aimed to evaluate the quality of yogurt samples prepared by commonly used probiotic starters in Iran, by analyzing the butyric acid content at different time intervals during consumption period. Based on the butyric acid concentrations the most suitable starter was identified.

## MATERIALS AND METHODS

### Starter cultures

Two commercially available lyophilized probiotic starter cultures for yogurt namely ABY1 and YO-MIX™ 211 were used for preparation of yogurt samples under laboratory conditions. ABY1 starter contained mixed cultures of *Lactobacillus acidophilus* LA-5, *Bifidobacterium* BB/2, *Streptococcus thermophilus* and *Lactobacillus delbruci* subspecies *bulgaricus*. YO-MIX™ 211 contained a mixture of *L.acidophilus, S.thermophilus*, *L. delbruci* subspecies *bulgaricus* and *Bifidobacterium lactis*.

### Preparation of Yogurt

Yogurt samples were prepared according to standard procedures. A litre of low fat milk (1.5% fat, 3% protein and approximately 10% non-fat dry powder) were added in a 3 liter stainless steel containers and heated at 90-95°C for 30 min. The yogurt mix was cooled to 42°C and inoculated with 0.04 gram of probiotic starters to achieve approximately 10^6^ cfu/g of the bacteria. The samples were mixed thoroughly and poured into 25ml sterile glass containers and allowed to stand for 2-3 hrs at 44°C. After coagulum was formed (pH 4.6) the samples were placed at refrigerated temperature (4°C). Control yogurt samples were obtained with similar protocols, with the addition of only ordinary dry yogurt starters containing a mixture of *S. thermophilus* and *L. delbrueckii* subsp *bulgaricus* only. These samples were and lacked the probiotic starters used in the preparation of probiotic yogurt samples. Overall six yogurt samples 2 from each (ABY1, 211 and ordinary yogurt) were prepared. Samples stored at 4°C were taken aseptically after 0, 1, 2, 10 and 20 days and analyzed for butyric acid concentrations by gas chromatography. Each experiment was performed in triplicate.

### Analysis of yogurt samples

Quantitative analysis of butyric acid and acetic acid in different yogurt samples stored at 4°C was performed by gas chromatography by the method of Ming-Hua et al 2001 (9) with slight modifications. Standard Butyric acid was obtained from Sigma (Germany), while all other chemicals and solvents used in the study were from Merck, (Germany).

### Trans-esterification and butyric acid extraction

Briefly, 2 grams of the yogurt samples were mixed with 100µl of 0.5 M methanolic NaOH and 2.5 ml of hexane in sample vials and shaken vigorously for 5 min using vortex mixer. 5µl of acetic acid standard was added to the homogenate and shaken thoroughly. Later, one gram of hydrophobic sodium sulphate salt was added and once more shaken vigorously for 1 min. The solution was allowed to stand for 1 hr, the upper phase (hexane) collected and the volume made up to 10 ml.

### Gas chromatography

Analysis was performed using a gas chromatograph (mod. 6890 Nλp) with DB-FFAP columns and connected to FID detector. Helium was used as the carrier and the make- up gas, with the flow rate of 1.3 ml/ min. The injection temperature was 250°C. The samples (1µl) were injected manually using the hot injector technique described earlier (10, 11).

### Calibration and Quantization

Six different concentrations of the standard butyric acid (0.1, 1, 2, 3, 5, and 7 M) were prepared and used for obtaining a calibration curve for quantization analysis.

### Statistical analysis

The data was subjected to analysis of variance (ANOVA,) using SPSS software with the formula: Y_ijk_= µ + α_j_ + ß_k_ + αß_jk_ + ɛ_ijk_

Where Y_ijk_ = observations, α _j_ = effect of samples, ß_k_ = effect of days, αß_jk_ = effect of samples x days, and ɛ_ijk_ = remaining effect

Differences were assessed based on Duncan test. P values < 0.05 were considered significant.

## RESULTS

Six yogurt samples including four probiotic and two without the probiotic starters were analyzed for butyric acid (C4) concentrations during storage at 4°C, at different time intervals. Gas chromatography was used in the study to measure the level of C4 derived from triglycerides by cold trans-esterification with sodium methylate.

**Fig. 1:**
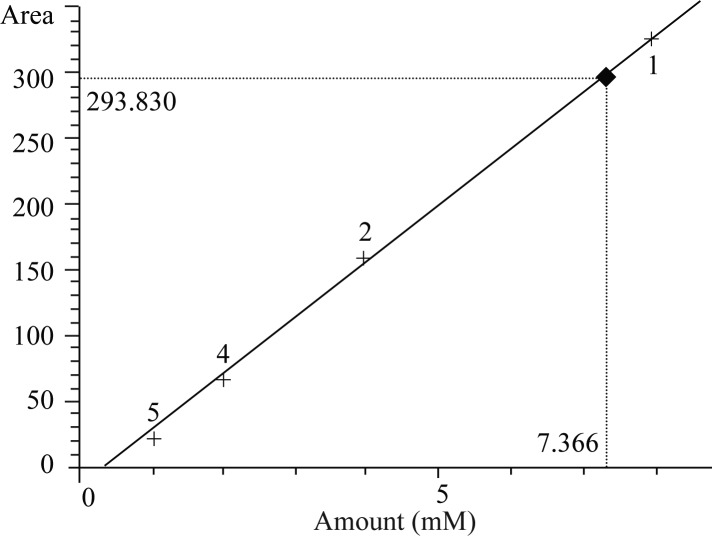
Calibration curve for standard butyric acid concentrations.

**Fig. 2:**
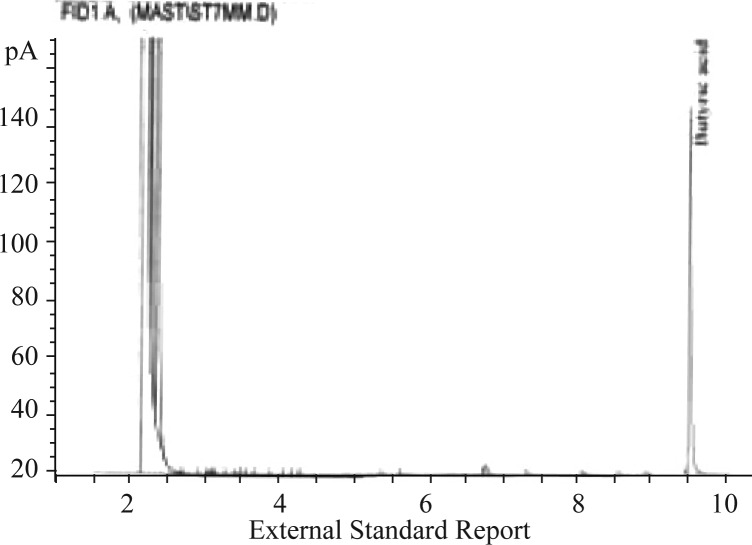
Chromatogram of standard butyric acid concentrations by gas chromatography.

In order to get quantitative results, a standard curve was drawn by injecting six different concentrations of butyric acid standards during analysis by gas chromatography (Fig. 1). The retention time of butyric acid is depicted in Fig. 2. According to the chromatogram the retention time at the applied conditions was 9.30 min.

**Fig. 3:**
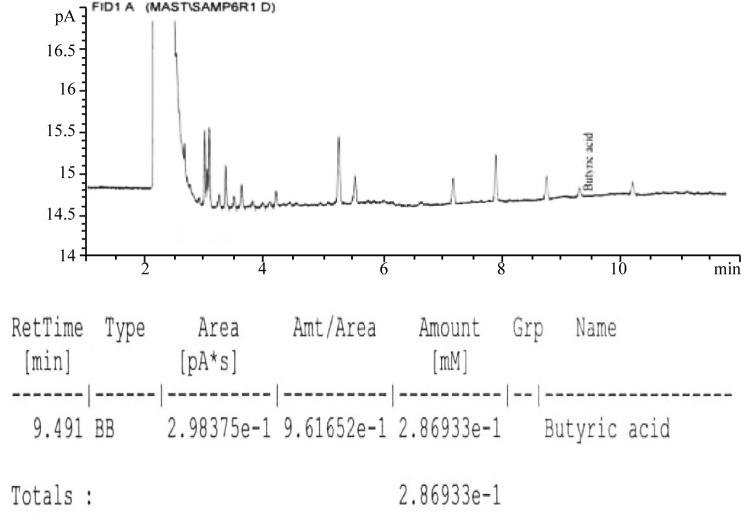
Chromatogram of yogurt samples.

Different levels of butyric acid were seen in the fats of yogurt samples tested at different time intervals at 4°C by gas chromatography (Table 1). Fig 3 shows the calibration curve and the chromatogram obtained after injecting different yogurt samples into the system. Compared to the ordinary yogurt samples which lacked the probiotic starter cultures, the butyric acid concentrations were higher in probiotic yogurt samples prepared with ABY1 and 211 starters, respectively.

**Table 1:** Percentage Butyric acid found in ordinary and probiotic yogurt fat analyzed at different time intervals by gas chromatography.

days	Ordinary yogurt			Probiotic yogurt samples		

	1 (%)	(%)2	211 (1) %	211 (2) %	ABY1 (1) %	ABY1 (2)%
First	0.06	0.07	0.177	0.167	0.2	0.202
Second	–	0.035	–	0.163	0.065	0.07
Tenth	0.067	0.01	–	0.178	0.075	0.02
Twentieth	–	0.025	–	–	–	0.063

The variance analyses related to butyric acid is as listed in Table 2. The differences owing to the significance of samples, time intervals (days), and sample into time interval were recorded. The mean difference between the samples based on Duncan test considering P < 0.05 is also seen in Tables 3 and 4. The results indicate significant differences in the level of butyric acid in ordinary and probiotic yogurt samples tested in the study.

**Table 2:** Analysis of variance related to butyric acid.

Source	TypeIII Sum of Squares	df	Mean Squares	F	Significance
Corrected model	6.94E-02^a^	10	6.945 E-03	12.816	0.003
Intercept	0.133	1	0.133	245.546	0.000
Sample	3.115 E-02	2	1.558 E-02	28.744	0.001
Days	1.341 E-02	3	4.471 E-03	8.250	0.015
Sample×days	1.464 E-02	5	2.928 E-03	5.403	0.032
Error	3.252 E-03	6	5.419 E-04		
Total	0.232	17			
Corrected total	7.270 E-02	16			

a R Squared = 0 .955 (Adjusted R Squared = 0.881)

**Table 3:** Comparison of samples average based on Duncan test.

Sample	N	Subset		
		1	2	3
Ordinary Yogurt	6	4.4500E-02		
ABY1	7		9.9429E-02	
211	4			0.17125
Significance		1.000	1.000	1.000

Sum of Squares ||| Means for groups in homogeneous subsets are displayed Based on Type

The error term is Mean Square (Error) = 5.419E-04

**Table 4:** Comparison of average storage time based on Duncan test.

Days	N	Subset	
		1	2
20^th^ day	2	4.4500E-02	
10^th^ day	5	7.0000E-02	
2^nd^ day	4	8.3250E-02	
1^st^ day	6		0.14600
Significance		0.075	1.000

Duncan

Means for groups in homogeneous subsets are displayed

Sum of Squares ||| Based on Type

The error term is Mean Square (Error) = 5.419E-04

The high R^2^ levels show high level of accuracy in the obtained results.

**Fig. 4:**
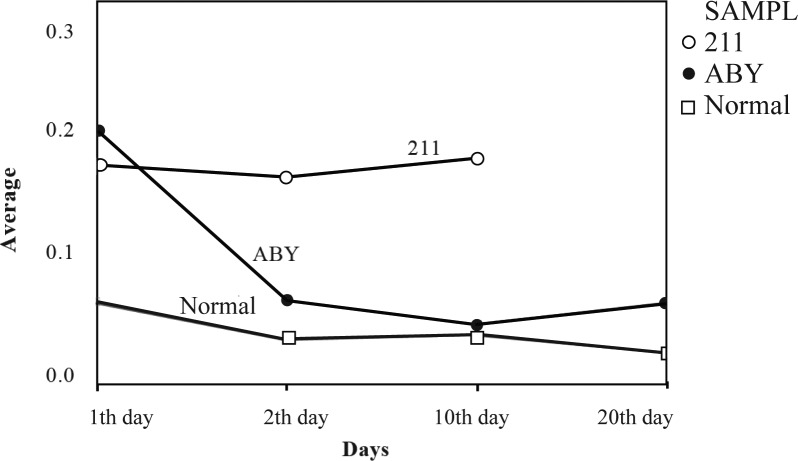
Reduction in butyric acid concentrations in different yogurt samples at different time intervals.

According to Fig. 4 the butyric acid concentrations in the tested yogurt samples during storage at different time intervals showed that the probiotic yogurt sample (211) had a reduction of approximately 0.16 % on first day of storage. The reduction slightly continued till day two, but later the level increased on day 10 reaching to the same levels as was on day two (0.17%).

The results of ABY1 samples were more significant and C4 levels in these samples were approximately 0.2% on first day which decreased significantly on day two (0.06%) and reached to 0.02% on day tenth. However, on day twentieth an increase of approximately 0.06% in the butyric acid concentrations was observed in this yogurt sample. Although the ordinary yogurt samples also showed levels of butyric acid concentrations till day twentieth (0.02% reduction), but the initial levels of C4 in these samples (0.06% reduction) were comparatively much lower than the levels in probiotic yogurt samples. The decrease in butyric acid concentrations in ordinary and probiotic yogurt samples was not similar at different time intervals examined.

## DISCUSSION

Butyric acid is one of the bioactive components of milk fat which has been known to reduce blood cholesterol levels and bowel infectious disease. Lactic acid bacteria in dairy products are involved in production of these free fatty acids (FFAs) including butyric acid and linoleic acid, by lipolysis of milk fats (12, 13, 14). The more of these probiotic bacteria, the more lactic, butyric and acetic acids are produced which increases the number of beneficial bacteria. Yogurt is known as the most famous carrier of these probiotics. Traditionally, *L. bulgaricus* and *Streptococcus thermophilus* have been the cultures used in yoghurt making. In recent years, some manufactures have added extra cultures to yogurt during processing to enhance its probiotic properties (6). The most often cultures added are *L. acidophilus, L. casei, L. reuteri* and *Bifidobacterium bifidum* (16, 17, 18). In this study we used two types of probiotic starters; ABY1 starter contained mixed cultures of *L. acidophilus* LA-5, *Bifidobacterium* BB/2, *S. thermophilus* and *L. delbruci* subspecies *bulgaricus*, YO-MIX™ 211 contained a mixture of *L.acidophilus, S. thermophilus*, *L. delbruci* subspecies *bulgaricus* and *Bifidobacterium lactis)*. The yogurt samples prepared with the above mentioned starters were then analyzed for their butyric acid concentrations at different time interval. Ordinary yogurt samples were prepared with the addition of ordinary dry yogurt starters (mixture of *S. thermophilus* and *L. delbrueckii* subsp *bulgaricus*).

Various methods for long-chain fatty acid exists which could also be applied to volatile short chain fatty acids ester (2, 10, 11), However, the measurement of the acid content derived from these reactions is very difficult due to their high volatility and their relative solubility in water. Fat hydrolysis method is also not a reliable method for obtaining free fatty acids as there is the possibility of losing volatile fatty acids by this method and hence we used trans-esterification method. The prepared yogurt samples (ordinary and probiotic) were subjected to trans-esterification before analyzing their butyric acid concentrations by gas chromatography.

With regard to the chemical structure of these fatty acids there are two proper analytical procedures for their determination, chromatography and electromigration (2, 19). Of the chromatographic methods, Gas chromatography (GC) is the most widely used method. In this study, we were able to identify butyric acid by using the GC system (using the method of trans-esterification instead of bleaching and heating). However the drawback of this method is that it does not provide simultaneous identification of other fatty acids. Fernandez *et al*. (20) also used two HPLC methods to isolate and determine the quality of the volatile organic acids. Later, Yang and his colleagues (11) reported GC method for detection of short chain organic acids including acetic, propionic, butyric, lactic acid in liquid foods.

Our results indicated increased levels of butyric acid concentrations in the probiotic yogurt samples prepared with microbial inoculation of ABY1 and 211 starters, compared to the control (ordinary yogurt) samples.

Beshkova and colleagues in 1998 (21) studied the carbonyl compounds and saturated fatty acids produced by pure cultures of *S. thermophilus, L. bulgaricus* and the Bulgarian yogurt starter cultures, during growth and storage at cold temperatures. Both the cultures showed significant levels of acetic, butyric and caproic acid. In another research conducted by Ogata and colleagues (22), yogurt samples inoculated with *B. Longum* (BB536) and ordinary yogurt samples fed to six healthy volunteers during two weeks, showed that Bifidobacterium containing yogurt was suitable for improving intestinal environment. A similar effect was also observed with ordinary yogurt samples, but to lesser extent than probiotic yogurt. Similarly, Adhikari and his colleagues (23), showed that the diversity of organic acids in fermented dairy foods is due to the activity of the added probiotic bacteria such as Bifidobacterium (*B. longum* B6 and ATCC15708). By using HPLC method, they showed that the concentrations of acetic and lactic acid increased during storage while uric and citric acid concentrations remained constant. However, no specific information was provided for butyric acid or propionic acid concentrations. In our study, the highest rate of butyric acid was observed in the yogurt samples with ABY1 starters on the first day which decreased gradually during storage but later on day twentieth it again increased. Several factors such as sample storage conditions, bacterial contamination or changes in the fermentation conditions could affect the levels of butyric acid (24).

Yadav and his colleagues (11), studied the production of fatty acids and conjugated linoleic acid (CLA) in the ordinary and probiotic yogurt dahi (prepared with buffalo milk) containing *L. acidophilus* and *L.casei*, during fermentation and after 10 days storage at 4°C. They reported that an increased level of fatty acids during fermentation and storage in the probiotic yogurt samples is mainly due the lypolysis of milk fat which was higher in the presence of probiotic bacteria. Compared to their results, the lower levels of butyric acid concentrations obtained in this study could also be attributed to lower milk fat levels of cow milk (1.5% fat yogurt prepared with cow milk) used in this study compared to buffalo milk.

Although the levels in the probiotic yogurt samples decreased initially but it was compensated at later phases and increased to recordable levels on day twentieth of storage. In contrast, in ordinary yogurt samples the reduction was continuous till day twentieth with no increase recorded in between.

In conclusion, considering the fact that volatile fatty acids including butyric acid has health benefits it is recommendable for the consumers to use probiotic yogurt which has higher levels of butyric acid with increased shelf life. Our results indicated that the commercially available probiotic starter ABY1 may be a suitable option for production of probiotic yogurt showing acceptable levels of butyric acid during storage.

However, insufficient viability and survival of these bacteria remain a problem in commercial food products. By selecting better functional probiotic strains and adopting improved methods to enhance survival, including the use of appropriate probiotic and the optimal combination of probiotics and prebiotics (symbiotic), an increased delivery of viable bacteria in fermented products to the consumers can be achieved.
